# BMI1 is downregulated by the natural compound curcumin, but not by bisdemethoxycurcumin and dimethoxycurcumin

**DOI:** 10.14814/phy2.12906

**Published:** 2016-08-22

**Authors:** Temitope A. Adeyeni, Natasha Khatwani, KayKay San, Uthayashanker R. Ezekiel

**Affiliations:** ^1^ Department of Biomedical Laboratory ScienceSaint Louis UniversitySt. LouisMissouri; ^2^ Department of Health Science and InformaticsSaint Louis UniversitySt. LouisMissouri

**Keywords:** Apoptosis, BMI1, colorectal cancer, phytochemical

## Abstract

The B‐cell‐specific Moloney murine leukemia virus integration site 1 (BMI1) locus encodes a 37‐kD protein that is a key regulatory component of the polycomb regulatory complex 1 (PRC1). When overexpressed in various cancer types, the BMI1 protein induces cell growth and promotes tumor growth in vitro and in vivo. Curcumin, a major phytochemical in turmeric (*Curcuma longa*), inhibits the proliferation and survival of many types of cancer cells, both in vitro and in vivo, and has been reported to reduce BMI1 expression in breast cancer cells. In this study, effects of curcumin and two analogs (bisdemethoxycurcumin and dimethoxycurcumin) on BMI1 expression were evaluated in DLD‐1 colorectal cancer cells. Bisdemethoxycurcumin (BDMC) is naturally occurring in turmeric, whereas dimethoxycurcumin (DMC) is a synthetic analog of curcumin. All three compounds reduced cell survival, but only the natural compound downregulated BMI1 protein expression; curcumin significantly reduced BMI1 levels more than bisdemethoxycurcumin and dimethoxycurcumin. In addition, curcumin and BDMC inhibit survival of the DLD‐1 colorectal cancer cells by inducing apoptosis, whereas DMC inhibits survival by a mechanism other than apoptosis.

## Introduction

Colorectal cancer is the third most common cancer among men and women in the United States (Siegel et al. 2013). Higher incidence rates were observed in people 40 years and older (Siegel et al. 2013). However, it was recently reported that there has been an increased incidence of colorectal cancer in the 20‐ to 34‐year‐old age group (Bailey et al. 2015). This was attributed to the western diet, which is high in processed foods and red meat but low in fruits and vegetables (Bailey et al. 2015).

The anticancer properties of fruits and vegetables may be partly due to their abundant phytochemicals (Srivastava et al. 2015). Phytochemicals refer to a variety of compounds that are produced by plants (Srivastava et al. 2015). Curcumin (CUR), the major phytochemical in turmeric (*Curcuma longa)*, has been shown to inhibit the proliferation of many types of cancers without creating adverse effects in patients taking oral doses up to 8–10 g per day (Hatcher et al. 2008; Shehzad et al. 2010; Carroll et al. 2011). Although CUR shows great potential as an anticancer drug, in vivo pharmacokinetic studies have shown it has low bioavailability (Carroll et al. 2011). However, some analogs of CUR are reported to have higher bioavailability. Two of these analogs were evaluated in this study — bisdemethoxycurcumin (BDMC) and dimethoxycurcumin (DMC). BDMC is naturally occurring in turmeric, whereas DMC is a synthetic CUR analog designed to have higher solubility and bioavailability than CUR (Anand et al. 2007; Shehzad et al. 2010; Hassan et al. 2015).

B‐cell‐specific Moloney murine leukemia virus integration site 1 (BMI1) encodes a 37‐kD polycomb group protein that serves as a key regulatory component of the polycomb regulatory complex 1 (PRC1) (Cao et al. 2011). The PRC1 complex is a chromatin structure modulator that normally regulates transcription of many important genes. BMI1 protein is involved in self‐renewal of adult stem cells (Cao et al. 2011). Overexpression of BMI1 in breast, prostate, lung, and ovarian cancers promotes the stem state of tumor cells and is associated with therapy failure (Cao et al. 2011). Reduction in BMI1 expression, however, leads to programmed cell death (apoptosis), senescence in tumor cells, and increased tumor cell susceptibility to cytotoxic agents and radiation therapy (Cao et al. 2011). Chowdhury et al. reported that acute myeloid leukemia patients with lower BMI1 expression levels have significantly longer overall survival, relapse‐free survival, and remission duration than patients with higher BMI1 expression levels (Chowdhury et al. 2007).

Guo et al. reported that CUR suppressed BMI1 protein expression in MDA‐MB‐231 and MDA‐MB‐435 breast cancer cells (Guo et al. 2013), but there are no studies that have reported such effects in other cancer cells. Thus, the objective of this present study was to assess the selected compounds’ (CUR, BDMC, DMC) effect on BMI1 expression in DLD‐1 colorectal cancer cells. Previously we identified that curcumin showed a similar inhibitory effect on genetically dissimilar DLD‐1, LoVo, and HCT‐116 cancer cell lines (Montgomery et al. 2016). Therefore, for this report, an exploratory study was done on the effect of the above three compounds on BMI1 expression using DLD‐1 cancer cells as a model system. We found that all three compounds inhibited survival of DLD‐1 cells. However, only curcumin did so by downregulation of BMI1 protein expression.

## Materials and Methods

### Chemical reagents and cell culture

Curcumin (purity >80%), bisdemethoxycurcumin (purity ≥98%), and dimethoxycurcumin (purity ≥90%) were purchased from Cayman Chemical Company (Ann Arbor, MI). All solutions were prepared as 100 mM stocks in DMSO and stored at −20°C. DMSO alone (0.2%) served as vehicle‐only control. DLD‐1 colorectal cancer cell isolates were purchased from the American Type Culture Collection (Manassas, VA). They were isolated from an adult male with Dukes’ type C colorectal adenocarcinoma (a subtype of colorectal cancer). Cells were grown at 37°C, 5% CO_2_ in DMEM (Sigma‐Aldrich, St. Louis, MO) supplemented with 10% FBS, penicillin/streptomycin, glutamine, sodium pyruvate, and HEPES buffer (Hyclone, Logan, UT).

### Cell survival studies

Five thousand DLD‐1 cells were seeded in each well of a 96‐well cluster plate (Sigma‐Aldrich, St. Louis, MO) and incubated for 12–16 h to allow adherence to the well surface. Cells were treated with varying concentrations (3.125–50 μmol/L) of CUR, BDMC, or DMC. Dilutions of each compound were prepared immediately before treatment, all treatments were performed in triplicates, and each experiment was performed at least three times. After addition of the phytochemicals, the cells were incubated for 48 h. Remaining live cells were fixed (1.1% glutaraldehyde, 15 min rotation, 1.2 ***g***) to the well surface, washed with water, dried, and then stained with crystal violet (0.1% crystal violet in 100 mmol/L MES, pH 6.0) (Van Schaeybroeck et al. 2005; Duessel et al. 2008; Jackson et al. 2008). Stained cells were solubilized in 10% acetic acid and absorbance at 562 nm was measured. Results were reported as percent cell survival compared to control.

### Cell viability and caspase‐3/7 activity

Five thousand cells were seeded in each well of a flat‐bottom, cell‐grade, 96‐well cluster black plate (BrandTech Scientific, Essex, CT) and incubated for 12–16 h before compound treatment. The cells were treated with three concentrations of each compound; these were half of each compound's half‐maximal inhibitory concentration (IC_50_), the IC_50_, and twice the IC_50_ (CUR: 6.25, 12.5, 25 μmol/L; BDMC: 5, 10, 20 μmol/L; and DMC: 1, 2, 4 μmol/L). All treatments were performed in triplicate and each experiment was performed three times.

At 48 h, the ApoToxGlo^™^ Triplex Assay was performed per manufacturer's instructions (Promega, Madison, WI). The assay measures fluorescence emitted by live and dead cells and also measures luminescent signals indicative of caspase‐3/7 activity. In this study, only fluorescent signals from live cells and luminescent signals from caspase‐3/7 activity were measured. In the assay, cell permeant, fluorogenic, peptide substrate (glycyl‐phenylalanyl‐aminofluorocoumarin; GF‐AFC) enter live cells, and are cleaved by proteases located in the cytoplasm. Fluorescence intensities, proportional to the number of live cells, were detected at the wavelengths 400_EX_/505_EM_ nm.

To measure caspase‐3/7 activity, a luminogenic substrate with attached tetrapeptide (DEVD: asp‐glu‐val‐asp) was added to the cells. Caspase digestion of the peptide releases a luciferase substrate known as aminoluciferin which is acted on by luciferase for production of luminescence: luminescence is proportional to caspase‐3/7 activity. All treatments were performed in triplicate, and each experiment was performed three times.

### BMI1 and apoptotic protein expression

Cells were treated with CUR (6.25–25 μmol/L), BDMC (6.25–25 μmol/L), or DMC (1.56–6.25 μmol/L) for 48 h. All treatments were performed in triplicate and each experiment was repeated three times.

Cells were lysed with radioimmunoprecipitation assay (RIPA) lysis buffer and supplemented with protease inhibitors (Thermo Scientific, Waltham, MA). Cell lysates containing 10 μg of protein were electrophoretically separated using SDS PAGE (4–12% gradient). The separated proteins were then electrophoretically transferred onto nitrocellulose membranes (Thermo Scientific, Waltham, MA).

B‐cell‐specific Moloney murine leukemia virus integration site 1, cleaved poly (ADP‐ribose) polymerase (PARP), procaspase‐3, and *β*‐actin (loading control) proteins were probed overnight with their respective monoclonal rabbit antibodies used at a 1:1000 dilution (Cell Signaling Technologies, Danvers, MA). Antirabbit IgG, horseradish peroxidase‐linked antibody (Cell Signaling Technology, Danvers, MA) at a 1:3500 dilution was used as secondary antibody. Signals were detected by chemiluminescence using SuperSignal West Pico Chemiluminescent Substrate (Thermo Scientific, Waltham, MA) and Thermo Fisher's myECL imager (Thermo Fisher Scientific, Danvers, MA). Even though the predicted molecular weight of BMI1 is 37 kDa, antibody (Cell Signaling Technology, Danvers, MA) recognizes the protein at 41 kDa in Western blots. The blots were stripped and reprobed with *β*‐actin. Immunoreactive band intensity of BMI1 was normalized by comparison to *β*‐actin signals. Fold differences were calculated with respect to vehicle control (0.2% DMSO)‐treated cells.

### Statistical analysis

All data were analyzed using one‐way analysis of variance (ANOVA) followed by a Student–Newman–Keuls post hoc analysis. All data were reported as mean ± SEM. Significance was reported as *P* < 0.05 unless otherwise noted.

## Results

### CUR, BDMC, and DMC decrease survival and viability of DLD‐1 cells

**Figure 1:**
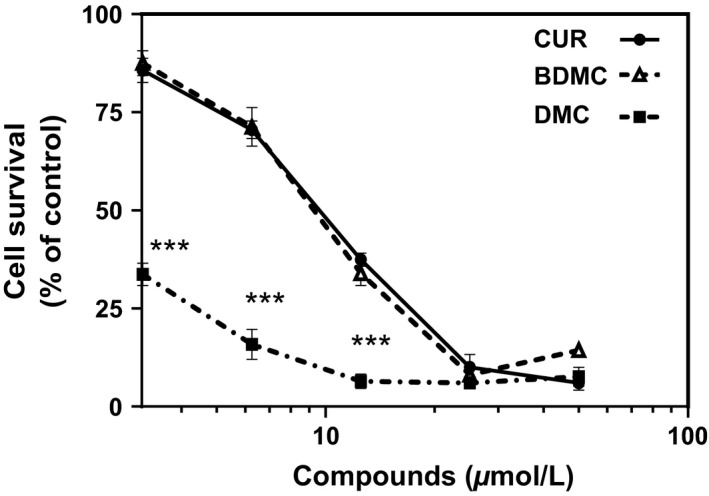
Effect of curcumin (CUR), bisdemethoxycurcumin (BDMC), and dimethoxycurcumin (DMC) on DLD‐1 colorectal cancer cell survival. DLD‐1 cells were cultured with various concentrations of CUR, BDMC, and DMC for 48 h. Survival of the cells (percent relative to control) was determined using the crystal violet method. All values represent the mean ± SEM of three independent experiments. ***P < 0.001 compared to CUR‐treated cells.

**Figure 2:**
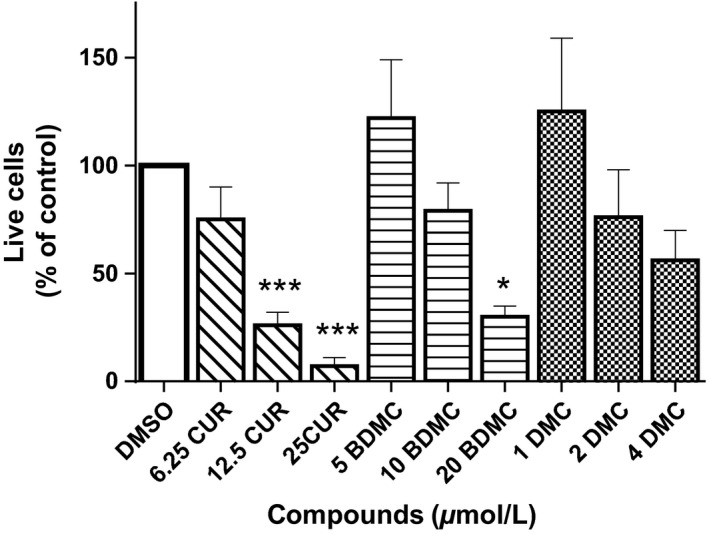
Effect of curcumin (CUR), bisdemethoxycurcumin (BDMC), and dimethoxycurcumin (DMC) on DLD‐1 colorectal cancer cell viability. DLD‐1 cells were cultured with three concentrations of each compound for 48 h. These were half of each compound's half maximal inhibitory concentrations (IC_50_), the IC_50_, and twice the IC_50_. Live cells were measured using ApoTox‐Glo^™^ Triplex assay (Promega, Madison, WI). Fluorescence (detected at 400_EX_/505_EM_ nm) is proportional to the number of live cells. All values represent the mean ± SEM of three independent experiments. ***P < 0.001 compared to vehicle control (0.2% DMSO‐treated cells). *P < 0.05 compared to vehicle control (0.2% DMSO‐treated cells).

We studied the effects of CUR, BDMC, and DMC on the survival of DLD‐1 colorectal cancer cells using the crystal violet method (Van Schaeybroeck et al. 2005; Duessel et al. 2008; Jackson et al. 2008). Cells were treated with varying concentrations of each compound (3.125–50 μmol/L) for 48 h. CUR, BDMC, and DMC all inhibit the survival of DLD‐1 colorectal cancer cells in a dose‐dependent manner (Fig. 1). However, inhibitory effects of DMC are significantly stronger than CUR and BDMC; it has an IC_50_ value of 1.90 ± 0.1 μmol/L. CUR and BDMC effects are not significantly different from one another with IC_50_ values of 9.83 ± 0.6 μmol/L and 8.39 ± 0.6 μmol/L, respectively. Cell viability measurements (ApoTox‐Glo^™^ Triplex assay, Promega, Madison, WI) showed that 48 h compound treatment (CUR: 6.25–25 μmol/L. BDMC: 5–20 μmol/L. DMC: 1–4 μmol/L) decrease the number of live cells in a dose‐dependent manner (Fig. 2). Based on previous studies (Yoon et al. 2014; Xu et al. 2015; Kim et al. 2016), concentrations of the three compounds used have no effect on cell viability of normal, noncancerous human cells. CUR (up to 40 μmol/L), BDMC (up to 80 μmol/L), and DMC (up to 30 μmol/L) showed no effect on viability of noncancerous cells (Yoon et al. 2014; Xu et al. 2015; Kim et al. 2016).

### CUR downregulates BMI1 protein expression

B‐cell‐specific Moloney murine leukemia virus integration site 1 is an oncogenic protein reported to be overexpressed in many cancers. Knockdown of BMI1 leads to apoptosis and senescence in tumor cells (Cao et al. 2011). Thus, we investigated whether CUR and its analogs (BDMC, DMC) could reduce BMI1 protein levels in DLD‐1 cells. Cells were treated with CUR (6.25, 12.5, 25 μmol/L), BDMC (6.25, 12.5, 25 μmol/L), and DMC (1.56, 3.13, 6.25 μmol/L) for 48 h. Western blot analysis of whole‐cell lysates from control (DMSO) and treated cells was performed. Band intensities of *β*‐actin (loading control) and BMI1 were determined by densitometry. BMI1 band density was normalized by comparison with *β*‐actin. Fold differences were calculated with respect to vehicle control (0.2% DMSO‐treated cells).

**Figure 3:**
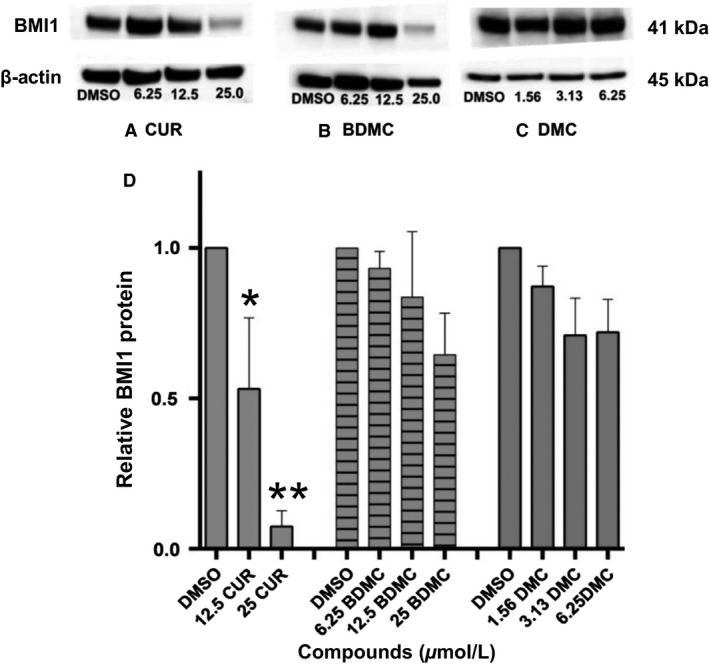
BMI1 protein expression in curcumin (CUR)‐, bisdemethoxycurcumin (BDMC)‐, and dimethoxycurcumin (DMC)‐treated DLD‐1 colorectal cancer cells. DLD‐1 cells were cultured with three concentrations of each compound for 48 h (CUR: 6.25, 12.5, 25 μmol/L. BDMC: 6.25, 12.5, 25 μmol/L. DMC: 1.56, 3.13, 6.25 μmol/L). Western blot analysis of whole‐cell lysates from vehicle control (0.2% DMSO) and compound‐treated cells, at 48 h, is shown ([A] CUR: 6.25, 12.5, 25 μmol/L; [B] BDMC: 6.25, 12.5, 25 μmol/L; [C] DMC: 1.56, 3.13, 6.25 μmol/L). *β*‐actin was used as loading control. (D) The relative intensity of BMI1 was evaluated as fold change compared to vehicle control (0.2% DMSO) after normalization with *β*‐actin. For curcumin, only 12.5 and 25 μmol/L band intensities were evaluated. All values represent mean ± SEM of three independent experiments. **P < 0.01 compared to vehicle control (0.2% DMSO‐treated cells). *P < 0.05 compared to 25 μmol/L CUR‐treated cells.

B‐cell‐specific Moloney murine leukemia virus integration site 1 expression analysis by Western blot indicates that only CUR decreases BMI1 protein levels compared to vehicle control (Fig. 3A). Figure 3D shows that there was a significant decrease between 25 μmol/L CUR and vehicle control (p < 0.01); there is also a significant decrease between 25 μmol/L CUR and 12.5 μmol/L CUR (*P* < 0.05). No significant decrease in BMI1 is shown by BDMC and DMC (Fig. 3B–D). These results indicate that only CUR suppresses BMI1 expression.

### CUR and BDMC induce apoptosis

**Figure 4:**
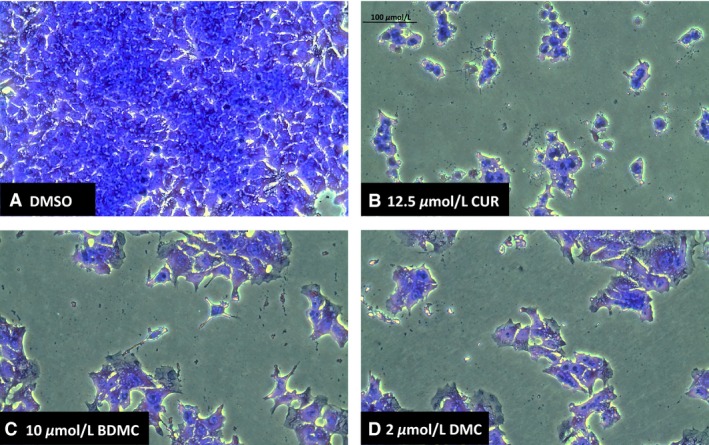
Microscopic images (20× magnification) of DLD‐1 colorectal cancer cells. Cells were cultured with half maximal inhibitory concentrations (IC_50_) of each compound for 48 h, fixed, and stained with crystal violet. (A) Vehicle control (0.2% DMSO); (B) 12.5 μmol/L curcumin (CUR); (C) 10 μmol/L bisdemethoxycurcumin (BDMC); and (D) 2 μmol/L dimethoxycurcumin (DMC).

Apoptosis or programmed cell death results in distinct cellular morphological changes and biochemical events including caspase‐3/7 activation, DNA fragmentation, chromatin condensation, and poly (ADP‐ribose) polymerase (PARP) cleavage (Tawa et al. 2004; Yoon et al. 2014). Cells treated with 12.5 μmol/L CUR, 10 μmol/L BDMC, or 2 μmol/L DMC show cell rounding and morphologic changes suggestive of apoptosis (Fig. 4A–D). Cells treated singly with CUR or BDMC show more pronounced rounding and blebbing than DMC‐treated cells. Additionally, we assessed apoptotic activity of the cells by measuring caspase‐3/7 activity.

**Figure 5:**
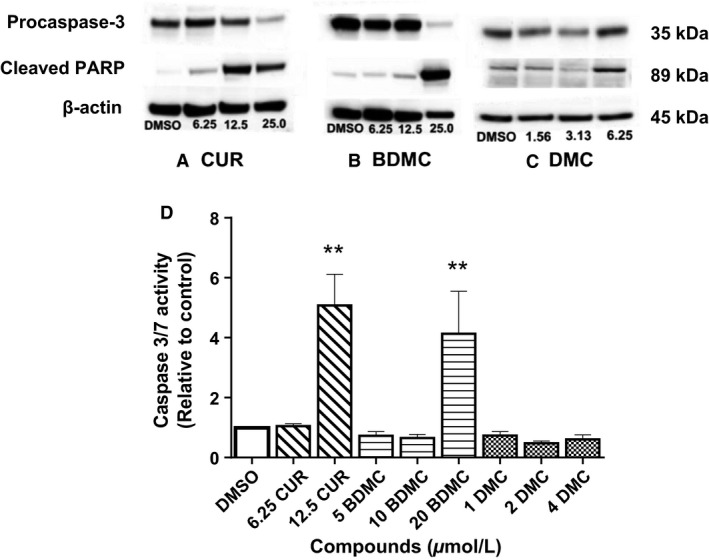
Apoptotic protein expression in curcumin (CUR)‐, bisdemethoxycurcumin (BDMC)‐, and dimethoxycurcumin (DMC)‐treated DLD‐1 colorectal cancer cells. DLD‐1 cells were cultured with three concentrations of each compound for 48 h (CUR: 6.25, 12.5, 25 μmol/L. BDMC: 6.25, 12.5, 25 μmol/L. DMC: 1.56, 3.13, 6.25 μmol/L). Western blot analysis of whole‐cell lysates from vehicle control and compound‐treated cells, at 48 h, is shown in (A) CUR, (B) BDMC, (C) DMC. Treated samples were probed overnight with procaspase‐3 and poly (ADP‐ribose) polymerase (PARP) antibodies. *β*‐actin was used as the loading control. (D) DLD‐1 cells were cultured with three concentrations of each compound for 48 h. These were half of each compound's half maximal inhibitory concentrations (IC_50_), the IC_50_, and twice the IC_50_. Luminescence emitted was proportional to caspase‐3/7 activity. **P < 0.01 compared to vehicle control. All values represent the mean ± SEM of three independent experiments.

We measured apoptosis by two methods: (1) an enzymatic method that measures caspase‐3/7; and (2) a Western blot method that detects active form of caspase‐3. PARP, which catalyzes poly(ADP) ribosylation of a variety of nuclear proteins, is cleaved by caspase‐3. To further confirm apoptotic progress in treated cells, we detected the presence of cleaved PARP by Western blot. Only 12.5 μmol/L CUR and 20 μmol/L BDMC significantly (*P* < 0.01) increased caspase‐3/7 activity (Fig. 5D). The 12.5 μmol/L CUR concentration shows 5‐fold more apoptosis and 20 μmol/L BDMC shows 4‐fold more apoptosis than control‐treated cells (Fig. 5D). DMC (1–4 μmol/L), however, does not significantly increase caspase‐3/7 activity (*n* = 3, *P* > 0.05) (Fig. 5D).

Western blot analysis was performed to detect protein markers of apoptosis, cleaved PARP, and caspase‐3. Activation of caspase‐3 leads to proteolytic cleavage of procaspase‐3 to active p17/p12 fragments (Tawa et al. 2004; Chaitanya et al. 2010). Therefore, a decrease in procaspase‐3 expression indirectly indicates that the cells underwent apoptosis (Tawa et al. 2004). Activated caspase‐3 cleaves PARP (116 kD) to produce an 89 kD cleaved product. PARP fragment that is cleaved indicates that the cells were undergoing apoptosis (Chaitanya et al. 2010). Western blot data show that CUR and BDMC increased cleaved PARP and decreased procaspase‐3 levels (Fig. 5A and B). DMC increases only cleaved PARP levels, whereas procaspase‐3 remains uncleaved (Fig. 5C). PARP is typically cleaved by caspase‐3; however, in DMC‐treated cells, cleaved PARP is evident in Western blot but there is no increase in caspase3/7 activity. This discrepancy indicates that an additional mechanism, such as necrosis, is generating cleaved PARP (Chaitanya et al. 2010). These findings indicate that CUR and BDMC inhibit the survival of DLD‐1 colorectal cancer cells by inducing apoptosis, whereas DMC may inhibit survival by alternative mechanisms.

## Discussion

Curcumin, bisdemethoxycurcumin, and dimethoxycurcumin inhibit the survival and viability of DLD‐1 colorectal cancer cells (Figs. 1, 2) in a dose‐dependent manner. Whereas the natural compounds (CUR and BDMC) exert similar inhibitory effects, the synthetic compound (DMC) exerts a stronger effect (Fig. 1). Interestingly, only CUR effects were mediated by the downregulation of the oncogenic protein BMI1 (Fig. 3) and the subsequent induction of apoptosis (Figs. 4, 5). BDMC and DMC did not affect the BMI1 levels but did affect cell viability. Additionally, we observed that CUR and BDMC, but not DMC, reduced cell survival by apoptosis indicating that inhibitory effects are mediated by alternative molecular mechanisms (Yoon et al. 2014).

Both CUR and BDMC exhibited apoptosis and the presence of active caspase‐3. Yoon et al. observed no morphological features of apoptosis in DMC‐treated breast cancer cell lines; they also found that DMC‐induced cell death is not inhibited by caspase‐8, ‐9, or ‐3 inhibitors (Yoon et al. 2014). This suggests that apoptosis is not involved in DMC‐induced cell death. Instead, they found that DMC significantly induces another form of programmed cell death, paraptosis (Yoon et al. 2014). Paraptosis is an alternative, nonapoptotic form of programmed cell death that lacks nuclear fragmentation, chromatin condensation, and formation of apoptotic bodies characteristic of apoptosis (Yoon et al. 2014). Markers of paraptosis, such as cytoplasmic vacuolation, were observed in both CUR‐ and DMC‐treated cells; however, DMC displayed stronger inhibitory and paraptotic effects than CUR (Yoon et al. 2014). The aforementioned study supports our observation that DMC does not significantly increase caspase‐3/7 activity (Fig. 5D) and does not induce the cleavage of procaspase‐3 (Fig. 5C). However, there remains no explanation for why cleaved PARP (a hallmark of apoptosis) was expressed in 6.25 μmol/L DMC‐treated cells (Fig. 5C), despite the fact that PARP is typically cleaved by caspase‐3 during apoptosis (Chaitanya et al. 2010).

Chaitanya et al. reported that when human leukemia Jurkat T cells were treated with inducers of apoptosis (150 μmol/L Vp‐16 or 150 nmol/L staurosporine) or necrosis (100 μmol/L HgCl_2_, 10% EtOH, or 0.1% H_2_O_2_), the cells expressed the same 89 kD fragment of PARP typically expressed only in apoptotic cells (Chaitanya et al. 2010). This was despite the fact that there was no apoptotic activity in the necrotic cells (Chaitanya et al. 2010). This observation is supported by reports of 5 μmol/L DMC's induction of both apoptotic and necrotic forms of cell death in MCF‐7 breast cancer cells (Kunwar et al. 2012). At higher doses (25 and 50 μmol/L), however, DMC primarily induces necrotic cell death (Kunwar et al. 2012). These findings explain why, in our study, DMC displays lower caspase‐3/7 activity than the other compounds (CUR, BDMC) and why the 89 kD cleaved PARP fragment is expressed even though procaspase‐3 is not cleaved (Fig. 5C). These studies show that DMC's inhibitory effects are driven by paraptotic and necrotic mechanisms (Kunwar et al. 2012; Yoon et al. 2014).

A low molecular weight compound, PTC‐209, induces a dose‐dependent inhibition of BMI1 expression in HCT‐116 human colorectal cancer cells and leads to cell death (Kreso et al. 2014). BMI1 targeted by PTC209 abrogates the tumorigenic capacity of colon cancer stem cells in vivo (Kreso et al. 2014). The aforementioned study highlights the importance of discovering compounds that target BMI1. Here, we found that the natural compound CUR inhibits BMI1 expression. Future studies will focus on understanding the mechanism of this inhibition.

Several studies report that *BMI1* gene expression can be regulated by microRNAs (miRNAs, miR). We hypothesize that CUR upregulates miRNAs that target *BMI1*. MiRNAs play a role in posttranscriptional gene expression and can act as tumor promoters or tumor suppressors (Teiten et al. 2010; Jansson and Lund 2012; Shah et al. 2012). In both cases, the target mRNA is inactivated/silenced (Jansson and Lund 2012). A single miRNA can have multiple mRNA targets (Teiten et al. 2010; Jansson and Lund 2012; Shah et al. 2012). There are several miRNAs that target BMI1 in various cancer types (miRNA‐ 218, 215, 200b, 200c, 128, 15a/b, 708, 16) (Bhattacharya et al. 2009; Saini et al. 2011; He et al. 2012; Liu et al. 2012; Sun et al. 2012; Guo et al. 2014; Jin et al. 2014; Yu et al. 2014; Jones et al. 2015).

When HCT‐116 and HT29 colorectal cancer cells are transfected with miRNA‐218, BMI1 gene and protein expression are significantly downregulated (He et al. 2012). A significant reduction in colony counts of the HCT‐116 and HT29 cell lines is also evident (He et al. 2012). The growth inhibitory effects have been further confirmed by tumorigenicity in vivo*,* where subcutaneous tumor growth is lower in pre‐miR‐218 transfected HCT‐116 cells compared with the control transfected HCT‐116 cells (He et al. 2012). Additionally, there is a significant increase in apoptotic cells and significant G2 cell‐cycle arrest (He et al. 2012).

Guo et al. (2013) showed that CUR inhibits MDA‐MB‐231 and MDA‐MB‐435 breast cancer cell survival and invasion. CUR's effect is caused by the upregulation of miRNA‐34a, which subsequently reduces BMI1 levels (Guo et al. 2013). This study demonstrates that CUR reduces BMI1 expression in breast cancer cells by upregulating miR‐34a (Guo et al. 2013). However, to the best of our knowledge, our study is the first to show that CUR reduces BMI1 expression in colorectal cancer cells. Therefore, our future studies will focus on understanding if CUR reduces BMI1 levels by upregulating one or more of the miRNAs shown to downregulate BMI1 expression (Bhattacharya et al. 2009; Saini et al. 2011; He et al. 2012; Liu et al. 2012; Sun et al. 2012; Guo et al. 2014; Jin et al. 2014; Yu et al. 2014; Jones et al. 2015).

## Conflict of Interest

None declared.
